# Intraoperative Application of Cold Atmospheric Plasma Reduces Inguinal Wound Healing Disorders—A Pilot Study

**DOI:** 10.3390/jcm14217533

**Published:** 2025-10-24

**Authors:** Ursula E. M. Werra, Wael Ahmad, Michael Schoepal, Tran T. Trinh, Bernhard Dorweiler

**Affiliations:** Department of Vascular and Endovascular Surgery, Faculty of Medicine, University Hospital Cologne, University of Cologne, 50937 Cologne, Germany; wael.ahmad@uk-koeln.de (W.A.); michael.schoepal@uk-koeln.de (M.S.); tran-trinh@uk-koeln.de (T.T.T.); bernhard.dorweiler@uk-koeln.de (B.D.)

**Keywords:** cold plasma, surgical site infection, intraoperative care, vascular surgery, wound revision, high-risk patients

## Abstract

**Background**: Inguinal wound healing disorders have been a relevant problem in the surgical treatment of peripheral arterial occlusive disease (PAD) for decades with reported rates of up to 30%. Despite the otherwise diverse innovations in vascular surgery, there are hardly any improvements in this area, on the contrary, comorbidities such as obesity, as relevant risk factors, continue to increase. The application of cold atmospheric plasma (CAP) has in turn shown promise in approaches for the treatment of chronic wounds, we therefore evaluated the potential reduction in inguinal wound healing disorders through the intraoperative application of CAP. **Methods**: We carried out a pilot study including 50 patients with a high risk for inguinal wound healing disorders that underwent a peripheral arterial reconstruction with inguinal access. Alternately, these patients were treated once intraoperatively with CAP (n = 25) or served as the control group (n = 25). The wound condition was then evaluated for the next fourteen days, with a follow up of three months. **Results**: The two groups showed no differences regarding risk factors such as smoking, obesity, PAD stage or surgery-related aspects like incision length or duration of surgery. No differences were found regarding wound-related readmission. However, the patients who had been treated intraoperatively with CAP showed a significant reduction in the need for surgical revisions due to inguinal wound healing disorders (8% vs. 32%, *p* = 0.034). **Conclusions**: This pilot study shows that the intraoperative use of CAP could be a promising approach to reduce major inguinal wound healing disorders.

## 1. Introduction

Approximately 237 million patients worldwide suffered from peripheral arterial occlusive disease (PAD) in 2015 [1]. In Germany alone, about 200,000 patients received inpatient treatment for PAD in 2018. And even if endovascular interventions are rising (about 275,000 interventions/year in 2018 in Germany), the total number of open surgical procedures also slightly increased over the years, reaching about 125,000 procedures/year in 2018 in Germany [2]. This is partly because in addition to improvements and innovations in the endovascular sector, surgical techniques have also developed further in recent years.

However, when discussing open surgical procedures in vascular surgery, wound healing disorders must always be considered. Despite the otherwise diverse innovations in vascular surgery, there are hardly any improvements regarding this topic up to today—with reported inguinal wound healing disorder rates up to 30% [3,4,5,6,7,8]. On the contrary, comorbidities, which harbour an intrinsic risk regarding wound healing such as diabetes mellitus, kidney failure or obesity, continue to increase [5,7,9]. Additionally, some patients show an even higher risk for wound healing disorders due to other factors like long procedural time (>240 min) or reoperations [9]. Besides possibly grave consequences for the patients themselves (e.g., higher rates of reoperation and amputation), wound healing disorders additionally lead to a relevant increase in health care costs (estimated for the United States: USD 12.000–38.000 per case) [7,8]. But even when following all well-known strategies, such as correct hair removal before surgery, single-shot antibiotics, incision at an oblique angle, atraumatic handling of tissue dissection, sparing of the lymph nodes and a careful wound closure technique, vascular surgery has not had its “breakthrough” moment regarding the prevention of SSIs, especially in the groin. The most promising approach today is the direct usage of prophylactic NPWT postoperatively [8].

Therefore, it has to be assumed that wound healing in these patients is generally intrinsically compromised, which would explain why previous approaches have only been partially successful.

Another wound entity characterised by compromised wound healing are chronic wounds. In recent years, the application of cold atmospheric plasma (CAP) has emerged as a promising approach here [10]. CAP, as a partially ionised gas, contains mainly RONS (reactive oxygen species), UV radiation, electromagnetic fields and charged particles. From in vitro experiments, the wide variety of effects of CAP on tissue are quite well known. Even if the mode of action is complex and not yet fully understood, the most relevant interactions could be allocated specifically. The reactive oxide and nitrogen species (RONS) especially play a major role regarding the proliferation of endothelial cells, the stimulation of angiogenesis or the increased distribution of growth factors like fibroblast growth factor 2 (FGF2) or vascular endothelial growth factor (VEGF). Besides that, RONS show bactericidal capacities through damaging of bacterial cell membranes by lipid peroxidation or inactivation of pathogens through damaging plasmid DNA or bacterial enzymes. UV radiation reduces hyperproliferation. Other aspects such as the improved adhesion and migration of cells (through the expression of integrins/catherins and type 1 collagen) are not fully understood yet [11,12].

This led to the idea of directly influencing the (patho-) physiological processes relevant in postoperative wound healing to avoid their deterioration. Accordingly, the question arises as to whether the usage of CAP could possibly reduce the development of inguinal wound healing disorders. As there is no data available to date, we carried out this pilot study to evaluate whether the intraoperative use of CAP could be an approach for the prevention of inguinal wound healing disorders in PAD patients by directly addressing the (patho-) physiology of wound healing.

## 2. Methods

This pilot study was carried out at the University Hospital Cologne, Cologne, Germany between September 2023 and April 2025.

### 2.1. Inclusion Criteria

Patients who underwent open surgery for PAD of the lower extremity with inguinal access and met the criteria for a high risk of developing a wound healing disorders were included in this study. Patients were considered high risk if they fulfilled the corresponding criteria of the score developed by Karl et al. 2013 [13]. This score (wound healing disorder score, WHD score) includes eleven factors. Four factors are weighted double (w.d.): diabetes mellitus (confirmed diagnosis and/or HbA1c > 6.5%) (w.d.), immunosuppression/steroid use (dosage above the Cushing threshold) (w.d.), terminal kidney failure (GFR < 15 mL/min/1.73 m^2^ or dialysis) (w.d.) and recurrent surgery/reoperation (open vascular reconstructive surgery on the same inguinal artery in medical history (w.d.). The other factors are PAD (Fontaine stage 2–4), active nicotine abuse, obesity (BMI > 30), age > 80, surgery lasting more than 4 h/240 min, intraoperative blood loss of more than 1.5 L and local radiotherapy and/or current chemotherapy (all single weighted) (Table S1). Added up, a patient was considered high risk if a score of 4 points or more was achieved.

### 2.2. Study Cohort

A total of 50 PAD patients who underwent surgical lower limb artery reconstruction (with inguinal access) and had a high risk of developing an inguinal wound healing disorder (score ≥ 4) were evaluated for this study.

A total of 25 patients were treated intraoperatively with argon-based CAP (kINPen^®^ MED, neoplas med GmbH, Greifswald, Germany) before standard cutaneous wound closure; the other 25 patients received standard-of-care treatment (no intraoperative CAP application and standard cutaneous wound closure). All wounds were then covered with a simple standard dressing. The assignment to the groups was performed alternatingly—this sequence was pre-defined: the allocation depended on the order of patient enrolment.

### 2.3. Plasma Application

**Figure 1:**
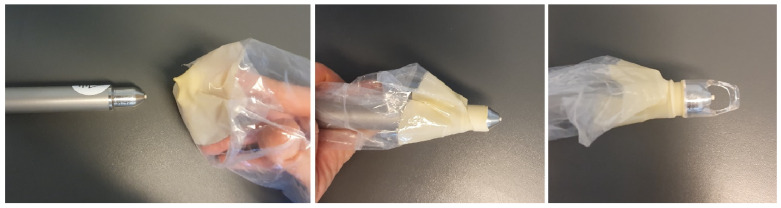
Sterile wrapping of the handpiece for intraoperative use: paediatric laparoscopic transducer cover and sterile conical spacer.

**Figure 2:**
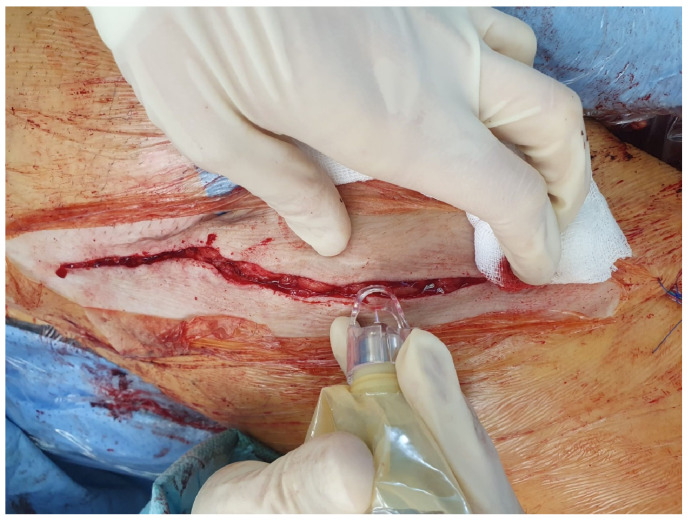
Right groin: intraoperative application of CAP to the wound edges of an inguinal incision (after subcutaneous sutures and directly before cutaneous closure). Covered with simple standard dressing afterwards.

CAP was applied intraoperatively to the inguinal wound edges, after suturing the subcutaneous tissue, directly before cutaneous closure (staples/sutures). For this purpose, the handpiece of the kINPen^®^ was wrapped sterilely using a paediatric laparoscopic transducer cover (Laparoscopic Transducer cover (15.2 × 244 cm), sterile, REF UA0067, CIVCO, Coralville, IA, USA) as well as the sterile conical spacer (Spacer (conical), sterile, REF KPM502, neoplas med, Greifswald, Germany) (Figure 1 and Figure 2).

The CAP was applied with a flow rate of 5 L/min at a constant pace of 5 mm per second. The sterile conical spacer ensured the correct distance to the wound edges. To approach the wound edges at a ≤45° angle, the wound edges were slightly lateralised/medialised manually, thus exposing the actual wound edge/the cutaneous incision surface (Figure 2).

### 2.4. Follow Up

Routine wound checks were carried out on the 2nd, 4th, 7th and 14th postoperative days and documented using a standardised wound documentation form. In addition to the length and shape of the incision and the suture material, aspects such as antibiotic treatment, quantity and type of wound secretion and fever as well as laboratory parameters such as leucocytosis or C-reactive protein were recorded. Pain was evaluated as local pain (regarding the wound itself) as well as general pain (assessed according to the numeric rating scale (NRS)). Reddening of the wound was also assessed: a plain reaction to staples (defined as localised reddening of the area surrounding the individual staple entry points without continuous reddening of the wound edge) was counted as no reddening. Continuous reddening of the wound edge(s) was measured as maximum spread of the reddening from the wound edge itself in millimetres. Additionally, the development of wound healing disorders (and severity (Szilagy classification [14])) as well as the need for a surgical revision (Szilagy Grades 2 and 3) were evaluated. The follow-up period was 3 months, at which point the patients were seen in the outpatient clinic for a last follow-up visit. The part of the study team that carried out the postoperative clinical assessment did not have information about whether a patient was in the treatment or control group.

The data was collected as part of the institute’s database ‘peripheral arterial vascular reconstructions’ (IRB approval of the University of Cologne was obtained, 22-1014).

### 2.5. Statistical Analysis

All statistical analyses were performed using SPSS Statistics V29 (IBM Corp., Armonk, NY, USA). Exploratory data analysis was conducted using appropriate inferential statistical methods. Categorical variables are presented as frequencies and percentages, while continuous variables are reported as medians with ranges.

Group differences were assessed using the Chi-square test, Fisher’s exact test or the Wilcoxon–Mann–Whitney U test, as appropriate according to the normality test. To examine the association between the use of cold atmospheric plasma (CAP) and the need for surgical wound revision, linear, ordinal and logistic regression models (both univariate and multivariate) were employed. A *p* value of <0.05 was considered statistically significant.

To mitigate selection bias and enhance group comparability, inverse probability weighting (IPW) based on propensity scores was applied for the analysis of the primary outcome.

The methodological steps included the following:

- Propensity Score Estimation:

Propensity scores were estimated via logistic regression using relevant baseline covariates (Table 1) to model the probability of receiving CAP treatment.

- Weighting:

Inverse probability weights were calculated to balance covariates between treatment groups, thereby improving comparability.

- Generalised Estimating Equations (GEEs):

GEE models were fitted using the weighted dataset to account for intra-group correlations and to provide robust standard errors. This approach reduces confounding and supports more reliable inferences regarding the treatment effect.

Results from the IPW-adjusted analyses are presented in Table 2 and Table 3.

## 3. Results

### 3.1. Demographics

Demographically, both groups were about equally characterised. The median age of patients in the plasma group (PG) was 69 (58–87) and in the control group (CG) 72; 16% (PG) and 24% (CG) of the patients were older than 80, respectively. Most patients were male.

The median score regarding risk of wound healing disorders (WHD score) in both groups was 5 (range: 4–7 PG, 4–8 CG, *p* = 0.463). A total of 64% of patients in the CG had diabetes mellitus (vs. 48%, *p* = 0.215) and 28% were obese with a BMI over 30 (vs. 8%, *p* = 0.138). A total of 12% of the patients in the PG were under treatment with immunosuppressants or steroids (vs. 0% CG, *p* = 0.235). Slightly more patients in the PG underwent recurrent surgery (68% vs. 48%, *p* = 0.252) or were active smokers (72% vs. 52%, *p* = 0.214). Only 4% of patients suffered from terminal kidney failure (both groups). Most patients in both groups were treated because of PAD Fontaine stage 2 (36% PG, 44% CG). Almost equally represented were patients with PAD Fontaine stage 3 (32% PG, 24% CG) and PAD Fontaine stage 4 (32% in both groups) (*p* = 0.940) (Table 1).

**Table 1:** Demographics and risk factor distribution. WHD score: risk of developing an inguinal wound healing disorder (Karl et al., 2013 [13]); PAD stage: peripheral arterial occlusive disease stage 2–4 (Fontaine stages).

(Risk) Factor	Plasma Group n = 25	Control Group n = 25	*p* Value
age	69 (58–87)	72 (61–89)	0.210
age > 80	4 (16%)	6 (24%)	0.725
gender			0.463
female	6 (24%)	3 (12%)	
male	19 (76%)	22 (88%)	
WHD score	5 (4–7)	5 (4–8)	0.215
diabetes mellitus	12 (48%)	16 (64%)	0.393
immunosuppression/steroid use	3 (12%)	0 (0%)	0.235
terminal kidney failure	1 (4%)	1 (4%)	1.00
recurrent surgery/reoperation	17 (68%)	12 (48%)	0.252
smoking	18 (72%)	13 (52%)	0.244
obesity (BMI > 30)	2 (8%)	7 (28%)	0.138
operative time > 4 h	16 (64%)	14 (56%)	0.773
blood loss > 1.5 L	0 (0%)	0 (0%)	
local radiation, chemotherapy	0 (0%)	0 (0%)	
PAD	25 (100%)	25 (100%)	
PAD stage	0.940		
2	9 (36%)	11 (44%)	
3	8 (32%)	6 (24%)	
4	8 (32%)	8 (32%)	

### 3.2. Surgery-Associated Risk Factors

Surgery-associated risk factors were also defined and compared between both groups. The median duration of the index surgery was 4.6 h in both groups (range 1.7–7.39 h PG, 1.75–9.85 h CG, *p* = 0.516). In both groups, staples were used in 92% of cases. In the other cases, single-button sutures (non-absorbable) were applied. The median incision length was 12 cm (8 cm–23 cm) for the PG and 12.5 cm (9 cm–19 cm) for the CG (*p* = 0.215). It was also assessed as to whether the incision avoided exceeding the inguinal fold (60% PG, 72% CG, *p* = 0.370) and was cranially lateralised (oblique incision) (88% in both groups).

The surgical procedures were categorised for their complexity: from the simpler procedure of a sole femoral reconstruction with or without thrombectomy of the iliac or femoral segment up to complex procedures such as crural bypasses with or without additional surgical measures. Most patients underwent procedures with the highest complexity score (4) (44% PG, 32% CG, *p* = 0.412). Additionally, the inguinal usage of prosthesis material (other than bovine) was evaluated. A distinction was made between “hybrid” grafts such as Omniflow^TM^ II (cross-linked ovine collagen with polyester mesh endoskeleton) and PTFE/Dacron grafts. In most cases (76% PG, 68% CG, *p* = 0.194) no prosthesis material other than bovine was used (Table 2).

**Table 2:** Surgery-associated risk factor distribution. Surgery complexity score: complexity of surgical procedure (1 = femoral reconstruction with/without thrombectomy, 2 = hybrid procedure (femoral reconstruction with pelvic and/or femoral endovascular intervention), 3 = bypass other than crural, 4 = crural bypass).

Surgery-Associated Risk Factor	Plasma Group n = 25	Control Group n = 25	*p* Value
incision length (cm)	12 (8–23)	12,5 (9–19)	0.215
oblique incision (cranially lateralised)	22 (88%)	22 (88%)	1.000
incision extending cranially beyond inguinal fold	15 (60%)	18 (72%)	0.370
suture material (staples)	23 (92%)	23 (92%)	1.000
duration of surgery (h)	4.6 (1.7–7.39)	4.6 (1.75–9.85)	0.516
surgery complexity score	0.412		
1	2 (8%)	6 (24%)	
2	8 (32%)	6 (24%)	
3	4 (16%)	5 (20%)	
4	11 (44%)	8 (32%)	
inguinal prosthesis material	0.194		
none	19 (76%)	17 (68%)	
Omniflow^TM^ II prosthesis	5 (20%)	3 (12%)	
PTFE/Dacron	1 (4%)	5 (20%)	

In order to take into account the aspect of procedures of varying complexity, we categorised procedures according to their complexity and assigned a score (surgery complexity score, Table 2). Sole femoral reconstructions (e.g., femoral thrombendartectomy with patchplasty) with or without thrombectomy were defined as procedures with a low complexity and a value of 1. Hybrid procedures (e.g., femoral reconstruction) combined with pelvic and/or femoral endovascular interventions were seen as intermediate complexity and assigned a value of 2. Bypasses were categorised as complex procedures; here a distinction was made between crural and all other bypasses. Bypasses other than crural were assigned the complexity value 3; crural bypasses were assigned the complexity value 4. There was no difference regarding the complexity of procedures between the two groups.

### 3.3. Wound Healing

Several aspects regarding wound healing were evaluated: the need for readmission to hospital due to wound complications, wound healing disorders not requiring surgical revision and wound healing disorders requiring surgical revision.

Wound healing aspects like local and general pain, redness, secretion volume (quantified as the need to change the dressing) or local swelling were evaluated for the first fourteen days. Leukocytes and CRP levels were also analysed. The two groups showed no significant differences regarding those parameters, with the exception of three aspects: the control group showed significantly more wound secretion at the 4th postoperative day (0.031) and also higher CRP levels at the 4th and 14th postoperative days (*p* = 0.040 and *p* < 0.001) (Table S2).

### 3.4. Wound Healing Disorders

Wound healing disorders were defined according to the Szilagy classification [7]: Grade 1: superficial wound healing disorder, only cutis affected. Grade 2: superficial, but subcutis also affected. Grade 3: deep wound healing disorder, arterial reconstruction affected.

No difference was found regarding superficial wound healing disorders (Szilagy Grade 1) (8% of patients in the PG and 4% in the CP) that could be managed conservatively. Significantly more patients in the CG needed a surgical revision as they developed a deep wound healing disorder of Szilagy Grade 2 or 3 (32% vs. 8%, *p* = 0.034). Surgical revisions were classified as minor or major revisions. Procedures in which the arterial reconstruction itself had to be revised were counted as major revisions. All other revisions (e.g., wound debridement and negative-pressure wound therapy) were counted as minor revisions (Table 3).

**Table 3:** Wound healing disorders: readmissions, Szilagy grading, need for surgical revision and antibiotic treatment.

Wound Healing Disorder	Plasma Group n = 25	Control Group n = 25	*p* Value
readmission to hospital due to wound complications	2 (8%)	6 (24%)	0.247
Szilagy Grade			0.034
2	1 (4%)	6 (24%)	
3	1 (4%)	2 (8%)	
surgical revision needed			0.034
minor revision	1 (4%)	6 (24%)	
major revision	1 (4%)	2 (8%)	
antibiotic treatment	2 (8%)	6 (24%)	0.247

There was no difference in PAD stages regarding the surgical revision group. A total of 50% suffered from PAD stage 4 and 50% from PAD stage 2 or 3. A total of 20% had undergone a recurrent procedure/reoperation.

Overall, 31% of PAD IV patients underwent surgical revision (vs. 14%, *p* = 0.234). Interestingly, of the patients who underwent a reoperation, only 6% needed an operative revision (vs. 38% *p* = 0.047).

There was no difference between PG and CG regarding readmission to the hospital due to wound complications (0.247). The median day at which readmission occurred was postoperative day 24 (9–32) in the control group and postoperative day 29 (17–42) in the plasma group.

### 3.5. Microbiological Findings

Microbiological samples from surgical revisions were positive for bacteria in 90% of cases.

In the control group, 50% of samples were positive for aerobe Gram-positive bacteria (*Streptococcus agalactiae*, *Streptococcus dysgalactiae*, *Staphylococcus epidermidis* and *Staphylococcus aureus*) and 75% for (facultative) anaerobe Gram-negative bacteria (*Proteus mirabilis*, *Klebsiella pneumoniae* and *Enterococcus faecalis*). In 37.5% of samples, multiple bacteria were detected. In two cases, the bacterial samples showed a switch over the duration of treatment to Candida spec., *Staphylococcus epidermidis*, *Enterococcus cloacae* and *Escherischia coli* or *Corynebacterium striatum*.

In the plasma group, 100% of samples were positive for aerobe Gram-positive bacteria: Staphylococcus aureus or Streptococcus epidermidis and Corynebacterium tuberculostaticum (mixed flora).

A total of 90% of patients undergoing surgical wound revision received antibiotic treatment: 55% were started on Piperacillin/Tazobactame, 11% received Ampicilline/Sulbactam as calculated antibiotic therapy and 33% received Amoxicillin/Clavulanic acid. In 77% of cases, the antibiotic treatment was then adapted to the microbiological findings over the course of treatment.

None of the patients developed fever. Overall, 24% of patients in the CG and 8% of the patients in the PG received antibiotic treatment (*p* = 0.247).

## 4. Discussion

Surgical site infections (SSIs) are significant complications after open surgery—increasing the duration of hospital stays and often requiring wound revision surgery or, even worse, the revision of the vascular reconstruction itself up to the explanation of vascular grafts and, worst of all, increasing the rate of major amputations and mortality [5,9].

Therefore, a solution to this problem has been searched for many years. Stewart et al. (2007) [15] carried out a meta-analysis of 34 randomised controlled trials regarding preventive measures of groin infections in vascular surgery. They found no effectiveness for rifampicin-bonded grafts, preoperative antiseptic skin baths, suction wound drainages, in situ bypass techniques, intraoperative glove change or different wound closure techniques. The only measure strongly supported by the literature was the prophylactic administration of antibiotics (single shot) preoperatively, which is part of the standards in perioperative vascular medicine today [15]. In 2023, Robbins et al. carried out a systematic review regarding preventative measures of groin incision surgical site infections. The preventive measures evaluated were Ioban adhesive drapes, prophylactic flaps, incision techniques, topical antibiotics and closed-incision negative wound therapy (ciNPWT). Overall, the evidence found was low, with ciNPWT representing the most promising approach, especially for high-risk patients. But it should be noted, however, that milder SSIs (Szilagy 1) in particular could be reduced here [7]. However, the difference between the ciNPWT approach and other measures is that it takes a wound-edge-related aspect into account: the tension from staples and sutures as they concentrate lateral tension to small points instead of the whole-wound edges. Additionally, it is proposed that the build-up of lymphatic fluid and therefore lymphocele formation could be decreased by ciNPWT systems as well as macerating of the wound edges. But reportedly, only up to about 10 mL of fluid was collected in the ciNPWT systems [8].

Focussing on the aspects of the resulting surgical wound itself is certainly the right approach here, as all well-established strategies such as incision strategies, atraumatic handling of tissue dissection, sparing of lymph nodes and careful wound closure focus on aspects regarding how the wound is formed and how macroscopic tissue damage can be avoided. And even if these are certainly not negligible, they do not seem to be sufficient to reduce SSI rates, as we are still talking about high-double-digit-percent rates. Therefore, there have to be underlying general pathophysiological factors that we have not managed to address yet that compromise wound healing, especially in this area, and it has to be assumed that these factors are aggravated by the multimorbidities of most PAD patients such as diabetes mellitus, smoking or obesity.

Already, in 1999, Raza et al. followed a very modern approach by addressing the problem by evaluating the skin perfusion/oxygenation after longitudinal groin incisions—a study that showed that skin oxygenation of the medial side of the wound was significantly lower than at the lateral side of the wound [16].

This indicates that a surgical incision in this area alone can jeopardise the complex interaction of haemostasis, inflammation, proliferation and dermal remodelling as the main aspects of wound healing. In this context, aspects such as angiogenesis, proliferation of endothelial cells, growth factors or the adhesion and migration of cells are of primary importance [12].

In this pilot study, the prophylactic intraoperative application of CAP directly to the cutaneous wound edges before closure of the skin showed a significant reduction in surgical revisions due to wound healing disorders. This positive impact can be considered a combination of bactericidal and direct healing-supporting effects: besides the damaging of bacterial cells and inactivation of pathogens, angiogenesis as well as adhesion/migration of cells is supported, among many other aspects. The approach is therefore the prevention of the deterioration of the (patho-) physiological processes contributing to postoperative wound healing. With regard to the certainly highly relevant bactericidal effect of CAP, the results of this pilot study once again confirm the in vitro results of Alkawareek et al. [17]: besides the overall strong bactericidal effect, bacteria from the Gram-negative spectrum are eliminated even more efficiently by CAP—especially if no biofilm formation prevails (e.g., in the case of an acute surgical wound). We found no Gram-negative species in the samples of the two cases from the plasma group that needed a surgical wound revision, whereas in 75% of surgical revised wounds in the control group, Gram-negative bacteria were present.

The intraoperative application is simple, takes a few minutes and apart from the argon gas that is used, only the spacer and the transducer cover are required as consumables. This is also an interesting approach from an environmental–economic point of view, as it does not use a huge amount of disposable materials.

The high revision rate of 32% in the control group can be explained by the fact that only patients with high-risk constellations for wound healing disorders were considered. This value is congruent with the literature—as expected, however, it corresponds to the upper range of the stated wound healing disorder rates due to the high-risk constellations of the patients [3,4,5,6,7,8].

Both groups showed no differences regarding risk factors such as age, diabetes mellitus, recurrent surgery, smoking or obesity. But in raw numbers, diabetes mellitus and obesity were observed more often in the control group while recurrent surgery and smoking were observed more often in the plasma group. Although these purely numerical differences are not statistically significant, they could influence the overall result with such a small sample size. Both groups had the same amount of PAD Fontaine stage 4 patients (32%)—patients that have an intrinsically higher risk of developing an SSI [18]. Surgery-associated risk factors were also comparable, with no differences in duration of surgery, rate of recurrent surgeries, incision length, complexity score or the usage of prosthesis material in the inguinal region. There were no differences regarding suture material, duration of surgery and form of incision between the groups. There were also no differences between the two groups in terms of antibiotic therapy. It can therefore be assumed that the positive effect shown can really be attributed to CAP, even though the study cohort is small.

For the treatment of chronic wounds, the effect of pain reduction was demonstrated for CAP in some studies [11]. Some individual patients in the PG (particularly those who underwent recurrent surgery) also reported less local pain comparably. Overall, a tendency towards less local pain sensation for the plasma group could be shown, but no significant difference was found between the groups.

## 5. Limitations

The informative value of this pilot study is intrinsically limited by the small number of patients. In addition, an allocation bias has to be assumed due to the lack of randomisation, which limits the conclusiveness of the results—even if the groups were well comparable in terms of the baseline risk factors. Both groups were treated within the same time period and by the same surgical team; therefore, a performance bias due to corresponding differences in operating techniques does not have to be assumed here.

## 6. Conclusions

In order to finally make progress on the critical issue of “inguinal wound healing disorder”, we need to focus on targeting underlying (patho-) physiological wound healing processes such as promotion of angiogenesis, cell adhesion/migration or collagen 1 expression and growth factor distribution. This pilot study shows that the intraoperative use of CAP could be a promising approach to achieve a reduction in deep inguinal wound healing disorders requiring surgical revision in high-risk patients. The bactericidal capacities of CAP and therefore the decontamination of the wound could especially play a pivotal role in this context. In order to further evaluate the use of CAP for the prevention of inguinal wound healing disorders—especially in high-risk patients—larger randomised multicentric studies should be carried out to verify the findings of this pilot study.

## Data Availability

The data that supports the findings of this publication is available from the corresponding author.

## Supplementary Materials

The following supporting information can be downloaded at: https://www.mdpi.com/article/10.3390/jcm14217533/s1, Table S1: Wound healing disorder score (T Karl, S. Woeste 2013 [13]); Table S2: Development of wound healing aspects over the first 14 postoperative days.

## References

[1] Song P., Rudan D., Zhu Y., Fowkes F.J.I., Rahimi K., Fowkes G.R., Rudan I. (2019). Global, regional, and national prevalence and risk factors for peripheral artery disease in 2015: An updated systematic review and analysis. Lancet Glob. Health.

[2] Kühnl A., Knipfer E., Lang T., Bohmann B., Trenner M., Eckstein H.-H. (2020). Krankenhausinzidenz, stationäre Versorgung und Outcome der peripheren arteriellen Verschlusskrankheit und arteriellen Thrombose/Embolie in Deutschland von 2005 bis 2018. Gefässchirurgie.

[3] Lee E.S., Santilli S.M., Olson M.M., Kuskowski M.A., Lee J.T. (2000). Wound infection after infrainguinal bypass operations: Multivariate analysis of putative risk factors. Surg. Infect..

[4] Kuy A., Dua An Desai S., Dua Ar Patel B., Tondravi N., Seabrook G.R., Brown K.R., Lewis B., Lee C.J., Kuy S., Subbarayan R. (2014). Surgical Site Infections after Lower Extremity Revascularization Procedures Involving Groin Incisions. Ann. Vasc. Surg..

[5] Turtiainen J., Saimanen E., Partio T., Kärkkäinen J., Kiviniemi V., Mäkinen K., Hakala T. (2010). Surgical Wound Infections after Vascular Surgery: Prospective Multicenter Observational Study. Scand. J. Surg..

[6] Rezk F., Åstrand H., Svensson-Björk R., Hasselmann J., Nyman J., Butt T., Bilos L., Pirouzram A., Acosta S. (2024). Multicenter parallel randomized trial evaluating incisional negative pressure wound therapy for the prevention of surgical site infection after lower extremity bypass. J. Vasc. Surg..

[7] Robbins J.M., Courtney J., Hingorani A. (2023). Systematic review of groin incision surgical site infection preventative measures in vascular surgery. J. Vasc. Surg..

[8] Matatov T., Reddy K.N., Doucet L.D., Zhao C.X., Zhang W.W. (2013). Experience with a new negative pressure incision management system in prevention of groin wound infection in vascular surgery patients. J. Vasc. Surg..

[9] Davis F.M., Sutzko D.C., Grey S.F., Mansour M.A., Jain K.M., Nypaver T.J., Gaborek G., Henke P.K. (2017). Predictors of surgical site infection after open lower extremity revascularization. J. Vasc. Surg..

[10] Stratmann B., Costea T.C., Nolte C., Hiller J., Schmidt J., Reindel J., Masur K., Motz W., Timm J., Kerner W. (2020). Effect of Cold Atmospheric Plasma Therapy vs Standard Therapy Placebo on Wound Healing in Patients with Diabetic Foot Ulcers: A Randomized Clinical Trial. JAMA Netw. Open.

[11] Werra U.E.M., Dorweiler B. (2023). Cold plasma in wound treatment—What do we know?. Gefässchirurgie.

[12] Barjasteh A., Kaushik N., Choi E.H., Kaushik N.K. (2023). Cold Atmospheric Pressure Plasma: A Growing Paradigm in Diabetic Wound Healing-Mechanism and Clinical Significance. Int. J. Mol. Sci..

[13] Karl T., Woeste S. (2013). Vermeidung von inguinalen Wundheilungsstörungen in der Gefäßchirurgie. Gefässchirurgie.

[14] Szilagyi D.E., Smith R.F., Elliott J.P., Vrandecic M.P. (1972). Infection in arterial reconstruction with synthetic grafts. Ann. Surg..

[15] Stewart A.H., Eyers P.S., Earnshaw J.J. (2007). Prevention of infection in peripheral arterial reconstruction: A systematic review and meta-analysis. J. Vasc. Surg..

[16] Raza Z., Newton D.J., Harrison D.K., McCollum P.T., Stonebridge P.A. (1999). Disruption of Skin Perfusion Following Longitudinal Groin Incision for Infrainguinal Bypass Surgery. Eur. J. Vasc. Endovasc. Surg..

[17] Alkawareek M.Y., Gorman S.P., Graham W.G., Gilmore B.F. (2014). Potential cellular targets and antibacterial efficacy of atmospheric pressure non-thermal plasma. Int. J. Antimicrob. Agents.

[18] Paius C.T., Constantin V.D., Carap A., Kretz B., Lhommet P., Gheorghiu R., Gaspar B., Epistatu D., Tarus A., Tinica G. (2022). Prediction and Management of Surgical Site Infections in Hybrid Vascular Surgery for Peripheral Artery Disease. Chirurgia.

