# Supplementary material for: Intraoperative Application of Cold Atmospheric Plasma Reduces Inguinal Wound Healing Disorders—A Pilot Study
Source: J Clin Med. 2025 Oct 24;14(21):7533. doi: 10.3390/jcm14217533 (PMC12608202; doi:10.3390/jcm14217533)
Supplement: Supplementary file 1 [file jcm-14-07533-s001.zip › jcm-3899322-supplementary.docx]

Table S1. Wound healing disorder score (T Karl, S. Woeste 2013)

diabetes mellitus 2

Immunosuppression/ steroid use 2

terminal renal insufficiency 2

recurrent surgery 2

smoking 1

obesity (BMI >30) 1

age >80 1

surgery >4h 1

blood loss >1,5l 1

local radiatio, chemotherapy 1

|  | 2nd p.o. day | |  | 4th p.o. day | |  | 7th p.o. day | |  | 14th p.o. day | |  |
| --- | --- | --- | --- | --- | --- | --- | --- | --- | --- | --- | --- | --- |
|  | PG | CG | p value | PG | CG | p value | PG | CG* | p value | PG*** | CG* | p value |
| general pain (NRS) | 1 (0-4) | 2 (0-7) | 0.992 | 0 (0-5) | 1 (0-5) | 0.329 | 1 (0-8) | 0 (0-6) | 0.332 | 0 (0-9) | 0 (0-3) | 0.479 |
| local pain | 5 (20%) | 6 (25%) | 0.733 | 3 (12%) | 7 (28%) | 0.144 | 4 (16%) | 6 (25%) | 0.496 | 3 (14%) | 4 (17%)** | 1.000 |
| primary healing | 25 (100%) | 25 (100%) |  | 25 (100%) | 25 (100%) |  | 100% | 24 (100%) |  | 22 (100%) | 21 (88%) | 0.235 |
| inpatient | 25 (100%) | 25 (100%) |  | 25 (100%) | 25 (100%) |  | 22 (88%) | 22 (91%) | 1.000 | 4 (18%) | 7 (29%) | 0.428 |
| reddening of wound edges |  |  | 0.317 |  |  | 0.927 |  |  | 0.342 |  |  | **0.043** |
| no/ reaction to staples | 24 (96%) | 25 (100%) |  | 24 (96%) | 24 (96%) |  | 23 (92%) | 20 (83%) |  | 19 (86%) | 14 (58%) |  |
| minor (<5mm) | 1 (4%) |  |  | 1 (4%) | 1 (4%) |  | 2 (8%) | 3 (13%) |  | 3 (14%) | 6 (25%) |  |
| major (>5mm) |  |  |  |  |  |  |  | 1 (4%) |  |  | 3 (13%) |  |
| NPWT |  |  |  |  |  |  |  |  |  |  | 1 (4%) |  |
| swelling (l) |  |  | 0.162 |  |  | 0.927 |  |  | 0.454 |  |  | 0.612 |
| no | 24 (96%) | 21 (84%) |  | 24 (96%) | 23 (92%) |  | 15 (60%) | 21 (88%) |  | 17 (77%) | 19 (79%) |  |
| mild | 1 (4%) | 4 (16%) |  | 1 (4%) | 2 (8%) |  | 10 (40%) | 3 (13%) |  | 4 (18%) | 4 (17%) |  |
| severe |  |  |  |  |  |  | 0 (0%) |  |  | 1 (5%) |  |  |
| NPWT |  |  |  |  |  |  |  |  |  |  | 1 (4%) |  |
| secretion |  |  | 0.136 |  |  | **0.031** |  |  | 0.40 |  |  | 0.865 |
| no | 23 (92%) | 19 (76%) |  | 23 (92%) | 20 (80%) |  | 24 (96%) | 18 (75%) |  | 18 (82%) | 19 (79%) |  |
| minimal | 1 (45) | 4 (16%) |  | 2 (8%) | 3 (12%) |  |  | 2 (8%) |  | 3 (14%) |  |  |
| dressing 1x/day | 1 (4%) | 2 (8%) |  |  | 2 (8%) |  | 1 (4%) | 3 (13%) |  | 1 (5%) | 2 (8%) |  |
| dressing 2x/day |  |  |  |  |  |  |  | 1 (4%) |  |  | 2 (8%) |  |
| NPWT |  |  |  |  |  |  |  |  |  |  | 1 (4%) |  |
| Leucocytes |  |  | 0.649 |  |  | 0.493 |  |  | 0.335 |  |  | 0.724 |
| normal | 19 (76%) | 18 (72%) |  | 21 (88%)* | 21 (91%)** |  | 18 (90%)° | 20 (95%)° |  | 16 (84%)°° | 12 (75%)°°° |  |
| >10.000 | 5 (20%) | 4 (16%) |  | 3 (13%)* | 2 (8%)** |  | 3 (15%) |  |  | 3 (16%)°° | 3 (19%)°°° |  |
| >15.000 | 1 (45) | 3 (12%) |  |  |  |  |  | 1 (5%)° |  |  |  |  |
| >20.000 |  |  |  |  |  |  |  |  |  |  | 1 (6%)°°° |  |
| CRP |  |  | 0.341 |  |  | **0.040** |  |  | **<0.001** |  |  | 0.110 |
| <5mg/l |  | 1 (4%) |  | 1 (4%)** |  |  | 1 (5%)° | 1 (5%)° |  | 4 (27%)°°°° | 1 (6%)°°° |  |
| >5mg/l | 11 (44%) | 9 (36%) |  | 12 (52%)** | 6 (26%)** |  | 19 (90%)° | 5 (24%)° |  | 8 (53%)°°°° | 8 (50%)°°° |  |
| >50mg/l | 8 (32%) | 4 (16%) |  | 6 (26%)** | 6 (26%)** |  | 1 (5%)° | 9 (42%)° |  | 2 (13%)°°°° | 5 (31%)°°° |  |
| >100mg/l | 4 (16%) | 5 (20%) |  | 3 (13%)** | 5 (22%)** |  |  | 5 (24%)° |  | 1 (6%)°°°° |  |  |
| >150mg/l | 1 (4%) | 3 (12%) |  | 1 (4%)** | 3 (13%)** |  |  |  |  |  | 2 (13%)°°° |  |
| >200mg/l | 1 (4%) | 1 (4%) |  |  | 3 (13%)** |  |  | 1(5%)° |  |  |  |  |
| >300mg/l |  | 2 (8%) |  |  |  |  |  |  |  |  |  |  |

Table S2: Development of wound healing aspects over the first 14 postoperative days. PG: plasma group, CG: control group, (l): local, (e): extremity, CRP: C- reactive protein, NPWT: negative pressure wound therapy, NRS: numeric rating scale, * one patient not evaluated, ** two patients not evaluated, *** three patients not evaluated, ° four patients not evaluated, °° six patients not evaluated, °°° nine patients not evaluated, °°°° ten patients not evaluated
